# Bleeding with intensive versus guideline antiplatelet therapy in acute cerebral ischaemia

**DOI:** 10.1038/s41598-023-38474-2

**Published:** 2023-07-20

**Authors:** Lisa J. Woodhouse, Jason P. Appleton, Hanne Christensen, Rob A. Dineen, Timothy J. England, Marilyn James, Kailash Krishnan, Alan A. Montgomery, Anna Ranta, Thompson G. Robinson, Nikola Sprigg, Philip M. Bath

**Affiliations:** 1 Stroke Trials Unit, Mental Health and Clinical Neuroscience, School of Medicine, South Block D Floor, Queen’s Medical Centre, University of Nottingham, Nottingham, NG7 2UH UK; 2 Stroke, Queen’s Medical Centre, Nottingham University Hospitals NHS Trust, Nottingham, NG7 2UH UK; 3 Bispebjerg and Frederiksberg Hospital, Department of Neurology, University of Copenhagen, Copenhagen, Denmark; 4 Radiological Sciences, Mental Health and Clinical Neuroscience, School of Medicine, Queens Medical Centre, University of Nottingham, Nottingham, NG7 2UH UK; 5 Derby Stroke Centre, University Hospitals of Derby and Burton, Derby, DE22 3NE UK; 6 Nottingham Clinical Trials Unit, Applied Health Research Building, School of Medicine, University of Nottingham, University Park, Nottingham, NG7 2RD UK; 7 Department of Medicine, University of Otago, Wellington, New Zealand; 8 Department of Neurology, Wellington Hospital, Wellington, New Zealand; 9 Department of Cardiovascular Sciences and NIHR Leicester Biomedical Research Centre, University of Leicester, Leicester, UK

## Abstract

Intensive antiplatelet therapy did not reduce recurrent stroke/transient ischaemic attack (TIA) events as compared with guideline treatment in the Triple Antiplatelets for Reducing Dependency after Ischaemic Stroke (TARDIS) trial, but did increase the frequency and severity of bleeding. In this pre-specified analysis, we investigated predictors of bleeding and the association of bleeding with outcome. TARDIS was an international prospective randomised open-label blinded-endpoint trial in participants with ischaemic stroke or TIA within 48 h of onset. Participants were randomised to 30 days of intensive antiplatelet therapy (aspirin, clopidogrel, dipyridamole) or guideline-based therapy (either clopidogrel alone or combined aspirin and dipyridamole). Bleeding was defined using the International Society on Thrombosis and Haemostasis five-level ordered categorical scale: fatal, major, moderate, minor, none. Of 3,096 participants, bleeding severity was: fatal 0.4%, major 1.5%, moderate 1.2%, minor 11.4%, none 85.5%. Major/fatal bleeding was increased with intensive as compared with guideline therapy: 39 vs. 17 participants, adjusted hazard ratio 2.21, 95% CI 1.24–3.93, *p* = 0.007. Bleeding events diverged between treatment groups in the 8–35 day period but not in the 0–7 or 36–90 day epochs. In multivariate analysis more, and more severe, bleeding events were seen with increasing age, female sex, pre-morbid dependency, increased time to randomisation, prior major bleed, prior antiplatelet therapy and in those randomised to triple vs guideline antiplatelet therapy. More severe bleeding was associated with worse clinical outcomes across multiple physical, emotional and quality of life domains.

*Trial registration* ISRCTN47823388.

## Introduction

The large Triple Antiplatelets for Reducing Dependency after Ischaemic Stroke (TARDIS) trial compared intensive versus guideline based antiplatelet therapy in patients with acute non-cardioembolic ischaemic stroke or transient ischaemic attack (TIA)^1^. Although intensive antiplatelet therapy (comprising combined aspirin, clopidogrel and dipyridamole) did not reduce stroke recurrence as compared with guideline therapy (combined aspirin and dipyridamole, or clopidogrel alone), a significant increase in bleeding, and fatal and major bleeding, was present with intensive antiplatelets. Overall, the trial was neutral and there was no net balance in favour of hazard or benefit^1^.

Long-term antiplatelet therapy is recommended by guidelines for patients with a history of ischaemic stroke or TIA. However, antiplatelet therapy is complicated by bleeding which may be minor (e.g. ecchymoses), severe causing intracranial haemorrhage or requiring blood transfusion, or fatal. The risk of bleeding is increased in certain race-ethnicity groups, and rises with age, and the number and type of antiplatelet agents that are taken^2^. Understanding the severity and causes of bleeding after acute cerebral ischaemia, including the impact of antiplatelet drugs, may help develop a more patient-centred approach to secondary prevention.

In this pre-planned secondary analysis of the TARDIS trial focussing on bleeding, we aim to assess: bleeding seen with intensive vs. guideline antiplatelet therapy; rates of bleeding; predictors of bleeding using the REACH, S_2_TOP-BLEED and intracranial-B_2_LEED_3_S^3^ scores^3–5^; and the relationship of bleeding with clinical outcomes after acute cerebral ischaemia.

## Results

### Baseline participant characteristics by bleeding events

Of the 3096 TARDIS participants, 3072 (99.2%) had data pertaining to presence or absence of bleeding events. Of these, 444 (14.5%) had a bleeding event during the trial period. Bleeding severity was distributed as: fatal 0.4%, major 1.5%, moderate 1.2%, minor 11.4% and none 85.5% (Table 1). In unadjusted univariate analyses, when compared to those with no bleeding event, those with a bleeding event were more likely to be female, have a higher baseline mRS, a longer time from onset to randomisation, a previous major bleed, a lower body weight, and be randomised to triple antiplatelet therapy (Table 2). Trends were observed across the severity of bleeding events for all the significant baseline characteristics in the above unadjusted univariate analyses, i.e. severe bleeding was especially related to these characteristics. Acute neuroimaging markers did not differ between those with and without bleeding events: mass effect was common affecting 44% and 45.9% of participants respectively; and median ASPECTS was 8^7,9^ (Table 3). Background neuroimaging markers also did not differ between those with and without bleeding events: cerebral atrophy in 93.4% and 91% respectively; leukoaraiosis in 43.1% and 41.9% respectively; and old stroke in 60%. The median ‘Brain frailty’ score was 2^1,3^, but did not differ between those with and without bleeding events. Of the bleeding scores, the S_2_TOP-BLEED scores were higher in those with any bleed compared to those with no bleeding events, but not across severity of bleeding events (Table 2).

**Table 1:** Severity of bleeding by on-treatment parameters.

	Bleeding^27^					
	Fatal	Major	Moderate	Minor	None	Trend p
Number of participants (%)	11 (0.4)	45 (1.5)	38 (1.2)	350 (11.4)	2628 (85.5)	–
Randomised antiplatelets (%)						
Aspirin, dipyridamole, clopidogrel	8 (0.5)	31 (2.0)	25 (1.6)	241 (15.6)	1236 (80.2)	< 0.001
Aspirin & dipyridamole	0 (0.0)	5 (0.7)	6 (0.9)	36 (5.2)	640 (93.2)	< 0.001
Clopidogrel	3 (0.4)	9 (1.1)	7 (0.8)	73 (8.6)	752 (89.1)	< 0.001
Adherence, first 7 days (%)						
First treatment	10 (0.4)	40 (1.6)	30 (1.2)	284 (11.1)	2203 (85.8)	0.37
All of first week	3 (0.1)	30 (1.4)	23 (1.1)	227 (10.7)	1831 (86.6)	0.010
Some of first week	11 (0.4)	45 (1.5)	37 (1.2)	346 (11.4)	2587 (85.5)	0.48
No treatment	0 (0.0)	0 (0.0)	1 (2.2)	4 (8.7)	41 (89.1)	0.48
Bleeding scores on treatment						
REACH^3^	10.8 (2.6)	10.8 (2.6)	10.6 (2.3)	10.3 (2.5)	10.2 (2.7)	0.17
S_2_TOP-BLEED^4^	13.5 (4.0)	13.4 (3.8)	13.2 (3.4)	12.6 (3.9)	11.6 (3.7)	< 0.001
Intracranial B_2_LEED_3_S^3^^5^	5.9 (1.6)	6.5 (1.8)	6.1 (1.4)	6.0 (1.8)	6.0 (1.8)	0.69

Percentages (%) are of the row total rather than the column total. Comparisons performed using Jonckheere-Terpstra test for binary data, and Kendall Rank Correlation for bleeding scores. Intracranial-B_2_LEED_3_S^3^: body mass index, blood pressure, lacune, elderly, ethnicity, coronary artery or cerebrovascular disease history, dual antithrombotic agent or oral anticoagulant, sex; REACH: Reduction of Atherothrombosis for Continued Health; S_2_TOP-BLEED:sex, smoking, blood pressure, lower body mass index, elderly, ethnicity, and diabetes.

**Table 2:** Baseline characteristics of the patients by bleeding and its severity.

	Bleeding			Bleeding^27^					
	Any	None	2p	Fatal	Major	Moderate	Minor	None	Trend p
Number of participants	444	2628	–	11	45	38	350	2628	–
Demographics									
Age (years)	69.8 (10.7)	68.8 (10.0)	0.054	73.7 (12.0)	69.6 (9.2)	72.1 (10.5)	69.5 (10.9)	68.8 (10.0)	0.072
Sex, male (%)	253 (57.0)	1681 (64.0)	0.0048	5 (45.5)	36 (80.0)	22 (57.9)	190 (54.3)	1681 (64.0)	0.0078
mRS, premorbid	0.0 [0.0, 0.0]	0.0 [0.0, 0.0]	0.014	0.0 [0.0, 0.0]	0.0 [0.0, 0.0]	0.0 [0.0, 0.0]	0.0 [0.0, 0.0]	0.0 [0.0, 0.0]	0.016
Time OTR [hr]	30.2 [23.1, 40.6]	29.1 [21.7, 39.4]	0.023	23.7 [15.3, 40.9]	28.7 [21.8, 42.1]	29.9 [19.0, 43.8]	30.4 [23.9, 40.2]	29.1 [21.7, 39.4]	0.026
Medical history (%)									
Smoking, current	103 (23.6)	675 (26.0)	0.29	4 (36.4)	10 (22.7)	6 (15.8)	83 (24.2)	675 (26.0)	0.28
Alcohol, heavy	39 (9.1)	250 (9.8)	0.64	1 (11.1)	5 (11.1)	7 (18.4)	26 (7.7)	250 (9.8)	0.72
Hypertension	264 (59.5)	1543 (58.7)	0.77	7 (63.6)	29 (64.4)	25 (65.8)	203 (58.0)	1543 (58.7)	0.71
Hyperlipidaemia	188 (43.2)	1118 (44.4)	0.64	2 (18.2)	24 (54.5)	17 (45.9)	145 (42.3)	1118 (44.4)	0.67
Diabetes mellitus	76 (17.1)	509 (19.4)	0.26	0 (0.0)	12 (26.7)	9 (23.7)	55 (15.7)	509 (19.4)	0.30
Atrial fibrillation	0 (0.0)	1 (0.0)	–	0 (0.0)	0 (0.0)	0 (0.0)	0 (0.0)	1 (0.0)	–
Stroke	46 (10.4)	300 (11.4)	0.52	0 (0.0)	7 (15.6)	3 (7.9)	36 (10.3)	300 (11.4)	0.52
Ischaemic heart disease	56 (12.6)	345 (13.1)	0.77	1 (9.1)	8 (17.8)	1 (2.6)	46 (13.1)	345 (13.1)	0.74
Peripheral artery disease	6 (1.4)	64 (2.5)	0.16	0 (0.0)	3 (6.8)	0 (0.0)	3 (0.9)	64 (2.5)	0.19
Bleed, major	7 (1.6)	12 (0.5)	0.0054	0 (0.0)	0 (0.0)	0 (0.0)	7 (2.0)	12 (0.5)	0.0083
Within 12 months	0 (0.0)	0 (0.0)	–	0 (0.0)	0 (0.0)	0 (0.0)	0 (0.0)	0 (0.0)	-
Clinical features (%)									
NIHSS (/42) †	2.7 (3.5)	2.8 (3.6)	0.59	4.1 (6.1)	3.9 (5.4)	3.0 (4.1)	2.5 (2.9)	2.8 (3.6)	1.00
OCSP, TACI ^23^	26 (5.9)	153 (5.8)	0.97	1 (9.1)	4 (8.9)	4 (10.5)	17 (4.9)	153 (5.8)	0.88
Systolic BP (mmHg)	142.9 (18.6)	143.6 (18.1)	0.43	158.9 (18.8)	144.7 (20.1)	148.8 (19.6)	141.5 (18.0)	143.6 (18.1)	0.45
Weight (approx. kg)	73.4 (18.5)	75.7 (16.3)	0.0076	70.0 (24.5)	73.9 (16.5)	73.9 (19.7)	73.3 (18.5)	75.7 (16.3)	< 0.001
Diagnostic (%)									
Qualifying event (%)									
Ischaemic stroke	315 (70.9)	1812 (68.9)	0.40	10 (90.9)	30 (66.7)	29 (76.3)	246 (70.3)	1812 (68.9)	0.38
TIA	129 (29.1)	816 (31.1)	0.40	1 (9.1)	15 (33.3)	9 (23.7)	104 (29.7)	816 (31.1)	0.38
Treatment (%)									
Prior Antiplatelet(s)	141 (31.8)	934 (35.5)	0.12	4 (36.4)	19 (42.2)	7 (18.4)	111 (31.7)	934 (35.5)	0.13
Prior Heparin	0 (0.0)	7 (0.3)	-	0 (0.0)	0 (0.0)	0 (0.0)	0 (0.0)	7 (0.3)	–
Alteplase	43 (9.7)	296 (11.3)	0.33	1 (9.1)	2 (4.4)	4 (10.5)	36 (10.3)	296 (11.3)	0.30
Post-event pre-randomisation Antiplatelet + alteplase	28 (6.3)	166 (6.3)	0.99	1 (9.1)	1 (2.2)	2 (5.3)	24 (6.9)	166 (6.3)	0.95
Randomised Antiplatelet									
ACD	305 (68.7)	1236 (47.0)	< 0.001	8 (72.7)	31 (68.9)	25 (65.8)	241 (68.9)	1236 (47.0)	< 0.001
Aspirin & dipyridamole	47 (10.6)	640 (24.4)	< 0.001	0 (0.0)	5 (11.1)	6 (15.8)	36 (10.3)	640 (24.4)	< 0.001
Clopidogrel	92 (20.7)	752 (28.6)	< 0.001	3 (27.3)	9 (20.0)	7 (18.4)	73 (20.9)	752 (28.6)	< 0.001
Imaging (%) (n = 2946) ‡									
Compatible lesion	166 (40.4)	1013 (42.1)	0.51	6 (60.0)	19 (44.2)	16 (44.4)	125 (38.8)	1013 (42.1)	0.57
Mass effect	73 (44.0)	465 (45.9)	0.64	2 (33.3)	8 (42.1)	5 (31.3)	58 (46.4)	465 (45.9)	0.60
ASPECTS [/10]	8.0 [7.0, 9.0]	8.0 [7.0, 9.0]	0.84	8.0 [6.0, 9.0]	7.0 [6.5, 8.0]	8.0 [6.0, 9.0]	8.0 [7.0, 9.0]	8.0 [7.0, 9.0]	0.79
Atrophy	384 (93.4)	2188 (91.0)	0.10	10 (100.0)	39 (90.7)	34 (94.4)	301 (93.5)	2188 (91.0)	0.10
Leukoaraiosis	177 (43.1)	1007 (41.9)	0.65	7 (70.0)	21 (48.8)	17 (47.2)	132 (41.0)	1007 (41.9)	0.57
Old stroke	247 (60.1)	1447 (60.2)	0.98	6 (60.0)	31 (72.1)	24 (66.7)	186 (57.8)	1447 (60.2)	0.93
Brain frailty score ^25^	2.0 [1.0, 3.0]	2.0 [1.0, 3.0]	0.54	2.0 [2.0, 3.0]	2.0 [2.0, 3.0]	2.0 [2.0, 3.0]	2.0 [1.0, 2.0]	2.0 [1.0, 3.0]	0.44
Bleeding scores									
REACH ^3^	8.2 (2.9)	8.1 (2.8)	0.35	9.0 (2.2)	8.5 (3.1)	8.2 (2.6)	8.2 (2.9)	8.1 (2.8)	0.34
S_2_TOP-BLEED ^4^	10.4 (3.6)	10.0 (3.2)	0.042	10.9 (3.2)	10.9 (3.7)	10.8 (3.2)	10.2 (3.6)	10.0 (3.2)	0.15
intracranial B_2_LEED_3_S^3^ ^5^	3.8 (2.2)	3.8 (2.1)	0.59	3.8 (1.6)	4.4 (2.1)	3.6 (1.9)	3.7 (2.2)	3.8 (2.1)	0.53

Data are number (%), median [interquartile range] or mean (standard deviation). 2p = difference between any and none bleeding events. Trend *p* = difference across the severity of bleeding events. † NIHSS was at the time of randomisation whether the index event was stroke or TIA; ‡ Adjudicated. TIA patients did not need a baseline scan prior to randomisation.

ACD: aspirin, clopidogrel, dipyridamole; ASPECTS: Alberta Stroke Program Early CT Score; BP: Blood Pressure; intracranial-B_2_LEED_3_S^3^: body mass index, blood pressure, lacune, elderly, ethnicity, coronary artery or cerebrovascular disease history, dual antithrombotic agent or oral anticoagulant, sex; mRS: modified Rankin scale; NIHSS: National Institutes of Health Stroke Scale; OCSP: Oxfordshire Community Stroke Project; OTR: onset to randomisation; PAD: peripheral artery disease; REACH: Reduction of Atherothrombosis for Continued Health; S_2_TOP-BLEED:sex, smoking, blood pressure, lower body mass index, elderly, ethnicity, and diabetes; TACI: total anterior circulation ischaemia; TIA: transient ischaemic attack.

**Table 3:** Bleeding by treatment groups.

Outcome	ACD	Clop	Asp + Dip	P	Trend p
Participants	1541	844	687		
*Primary safety*					
Ordinal bleeding (%)	305 (19.8)	92 (10.9)	47 (6.8)	< 0.001	< 0.001
Fatal^38^	8 (0.5)	3 (0.4)	0 (0.0)	–	–
Major	31 (2.0)	9 (1.1)	5 (0.7)	–	–
Moderate	25 (1.6)	7 (0.8)	6 (0.9)	–	–
Minor	241 (15.6)	72 (8.5)	36 (5.2)	–	–
None	1236 (80.3)	752 (89.2)	640 (93.2)	–	–
*Sensitivity analyses*					
Fatal or major^38^	39 (2.5)	12 (1.4)	5 (0.7)	0.0065	0.0019
Bleeding (%)					
Intracranial bleeding	16 (1.0)	4 (0.5)	1 (0.1)	0.044	0.012
Intracerebral	13 (0.8)	3 (0.4)	1 (0.1)	0.099	0.025
Subdural or extradural	2 (0.1)	0 (0.0)	0 (0.0)	0.72	0.18
Fatal	6 (0.4)	3 (0.4)	0 (0.0)	0.30	0.17
Major	9 (0.6)	0 (0.0)	1 (0.1)	0.030	0.027
Fatal or major	15 (1.0)	3 (0.4)	1 (0.1)	0.040	0.011
Extracranial	293 (19.0)	89 (10.5)	46 (6.7)	< 0.001	< 0.001
Gastrointestinal	48 (3.1)	20 (2.4)	14 (2.0)	0.30	0.11
Other	255 (16.5)	71 (8.4)	33 (4.8)	< 0.001	< 0.001
Fatal	2 (0.1)	0 (0.0)	0 (0.0)	0.72	0.18
Major	24 (1.6)	9 (1.1)	4 (0.6)	0.14	0.047
Fatal or major	26 (1.7)	9 (1.1)	4 (0.6)	0.084	0.025
Stroke or major haemorrhage	87 (5.6)	38 (4.5)	31 (4.5)	0.38	0.18
MACE or major haemorrhage	102 (6.6)	53 (6.3)	45 (6.6)	0.95	0.87

Data are number (%); comparison of three groups by Chi-square/Fisher’s exact test for direct comparisons or the Jonckheere-Terpstra test for trend. Haemorrhage is most severe, not first, bleed over 90 days. No subarachnoid haemorrhages occurred.

ACD: aspirin, clopidogrel and dipyridamole; Asp: aspirin; Clop: clopidogrel; Dip: dipyridamole; MACE: major adverse cardiovascular event.

Adjusted univariate associations between baseline variables and ordinal bleeding revealed that increasing age, female sex, increasing pre-morbid mRS, longer time from onset to randomisation, prior major bleed, and lower body weight were all associated with more severe bleeding events (Table 4). In adjusted multivariate models, all these baseline variables except body weight maintained statistical significance. Randomisation to triple antiplatelet therapy was associated with more severe bleeding in both univariate and multivariate analyses. Prior antiplatelet therapy was associated with more severe bleeding in multivariate but not univariate models. There were no significant associations between acute and background imaging markers and ordinal bleeding events. The S_2_TOP-BLEED score at baseline was associated with more severe bleeding in both univariate and multivariate analyses, whilst the S_2_TOP-BLEED score on treatment was associated with more severe bleeding in univariate but narrowly missed statistical significance in the multivariate model. There were no significant associations noted for other bleeding scores (Table 4).

**Table 4:** Univariate and multiple variable associations between baseline variables and ordinal bleeding.

	Univariate ß or OR	*p*	Multivariate ß or OR	*p*
Demographics				
Age (years)	1.01 (1.00, 1.02)	0.045	1.01 (1.00, 1.02)	0.044
Sex, male (%)	0.76 (0.62, 0.93)	0.0080	0.79 (0.64, 0.98)	0.033
mRS, premorbid	1.25 (1.04, 1.51)	0.019	1.28 (1.05, 1.57)	0.017
Time OTR [hr]	1.01 (1.00, 1.02)	0.028	1.01 (1.00, 1.02)	0.037
Medical history (%)				
Smoking, current	0.88 (0.69, 1.11)	0.28	0.90 (0.69, 1.17)	0.43
Alcohol, heavy	0.94 (0.66, 1.34)	0.72	0.98 (0.67, 1.42)	0.90
Hypertension	1.04 (0.85, 1.27)	0.71	1.10 (0.87, 1.38)	0.42
Hyperlipidaemia	0.96 (0.78, 1.17)	0.67	1.05 (0.83, 1.33)	0.69
Diabetes mellitus	0.87 (0.67, 1.13)	0.30	1.22 (0.88, 1.69)	0.23
Stroke	0.90 (0.65, 1.25)	0.52	0.97 (0.67, 1.41)	0.89
Ischaemic heart disease	0.95 (0.70, 1.29)	0.74	1.01 (0.72, 1.43)	0.95
Peripheral artery disease	0.57 (0.24, 1.32)	0.19	0.59 (0.25, 1.41)	0.24
Bleed, major	3.04 (1.23, 7.47)	0.016	3.07 (1.19, 7.91)	0.020
Clinical features				
NIHSS †	0.99 (0.97, 1.02)	0.71	0.99 (0.95, 1.03)	0.56
OCSP, TACI	1.03 (0.67, 1.58)	0.88	0.95 (0.52, 1.75)	0.88
Systolic BP (mmHg)	1.00 (0.99, 1.00)	0.55	1.00 (0.99, 1.01)	0.69
Weight (approx. kg)	0.99 (0.99, 1.00)	0.0078	1.00 (0.99, 1.00)	0.43
Diagnostic				
Qualifying event, ischaemic stroke (%)	1.10 (0.89, 1.38)	0.38	1.19 (0.89, 1.60)	0.25
Treatment				
Prior antiplatelet(s)	0.85 (0.68, 1.05)	0.13	64.65 (3.01, 1387.12)	0.0077
Alteplase	0.84 (0.60, 1.17)	0.30	0.71 (0.49, 1.02)	0.062
Post-event pre-randomisation antiplatelet(s) + alteplase	0.99 (0.65, 1.49)	0.95	1.39 (0.70, 2.75)	0.35
Randomised antiplatelet				
Aspirin, dipyridamole, clopidogrel	2.46 (1.99, 3.05)	< 0.001	2.55 (2.05, 3.17)	< 0.001
Aspirin & dipyridamole	0.37 (0.27, 0.51)	< 0.001	0.59 (0.38, 0.92)	0.019
Clopidogrel	0.65 (0.51, 0.83)	< 0.001	1.68 (1.09, 2.60)	0.019
Imaging (n = *2946) ‡*				
Lesion compatible with presentation	0.94 (0.76, 1.16)	0.57	0.92 (0.73, 1.16)	0.46
Mass effect	0.91 (0.66, 1.27)	0.60	0.86 (0.60, 1.23)	0.40
ASPECTS [/10]	0.98 (0.87, 1.10)	0.68	0.98 (0.86, 1.12)	0.80
Atrophy	1.41 (0.93, 2.13)	0.11	1.53 (0.98, 2.37)	0.059
Leukoaraiosis	1.06 (0.86, 1.31)	0.57	0.97 (0.77, 1.22)	0.78
Old stroke	1.01 (0.82, 1.25)	0.93	1.00 (0.80, 1.25)	0.99
Brain frailty score^25^	1.07 (0.94, 1.22)	0.33	1.04 (0.90, 1.21)	0.60
Bleeding scores				
REACH				
At baseline	1.02 (0.98, 1.06)	0.32	1.01 (0.96, 1.08)	0.64
On treatment	1.03 (0.99, 1.07)	0.14	0.94 (0.88, 1.01)	0.090
S_2_TOP-BLEED score				
At baseline	1.03 (1.00, 1.07)	0.033	1.07 (1.01, 1.14)	0.021
On treatment	1.09 (1.06, 1.12)	< 0.001	1.07 (1.00, 1.16)	0.066
Intracranial B_2_LEED_3_S^3^ score				
At baseline	0.99 (0.94, 1.04)	0.65	1.02 (0.95, 1.09)	0.63
On treatment	1.01 (0.96, 1.07)	0.66	0.99 (0.91, 1.06)	0.73

Data are number (%) and odds ratio or ß with 95% confidence intervals. Comparisons by binary logistic regression, ordinal logistic regression or multiple linear regression models with adjustment for baseline factors. Stroke/TIA is given by severity; where a patient had more than one event over 90 days, the most severe event is used.

ASPECTS: Alberta Stroke Program Early CT Score; BP: blood pressure; Intracranial-B_2_LEED_3_S^3^: body mass index, blood pressure, lacune, elderly, ethnicity, coronary artery or cerebrovascular disease history, dual antithrombotic agent or oral anticoagulant, sex; mRS: modified Rankin scale; NIHSS: National Institutes of Health Stroke Scale; OCSP: Oxfordshire community stroke project; OR: odds ratio; OTR: onset to randomisation; REACH: Reduction of Atherothrombosis for Continued Health; S_2_TOP-BLEED:sex, smoking, blood pressure, lower body mass index, elderly, ethnicity, and diabetes; TACI: total anterior circulation ischaemia.

### Bleeding by on-treatment parameters

**Figure 1:**
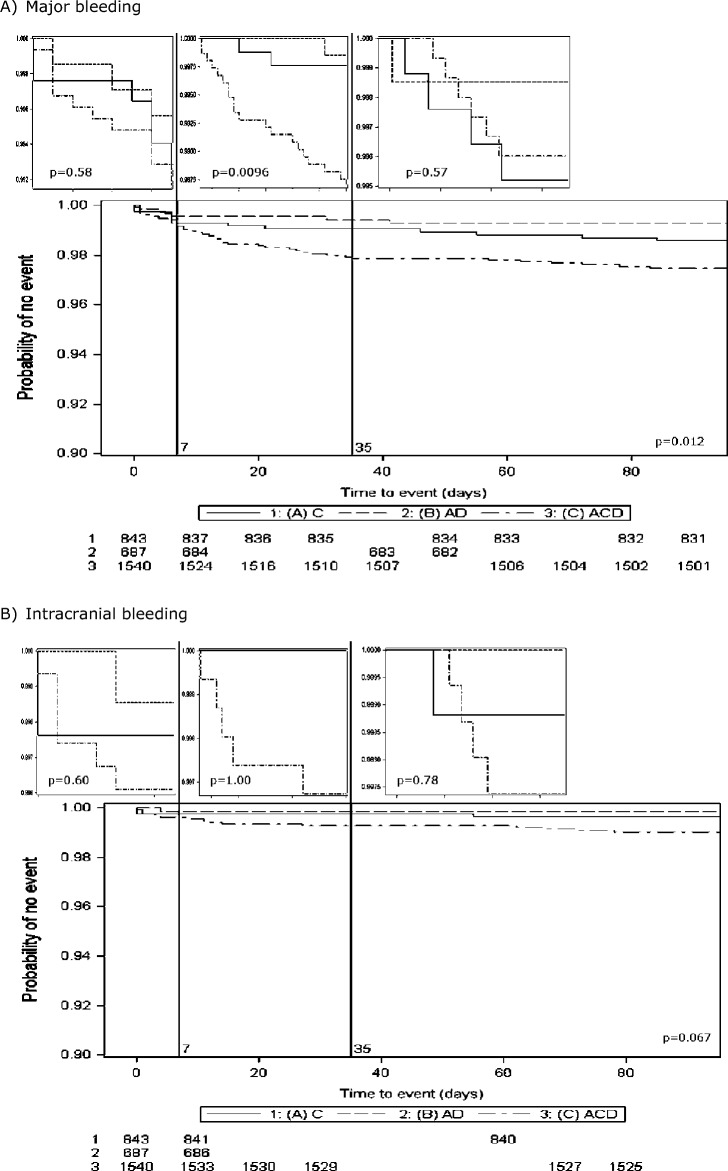

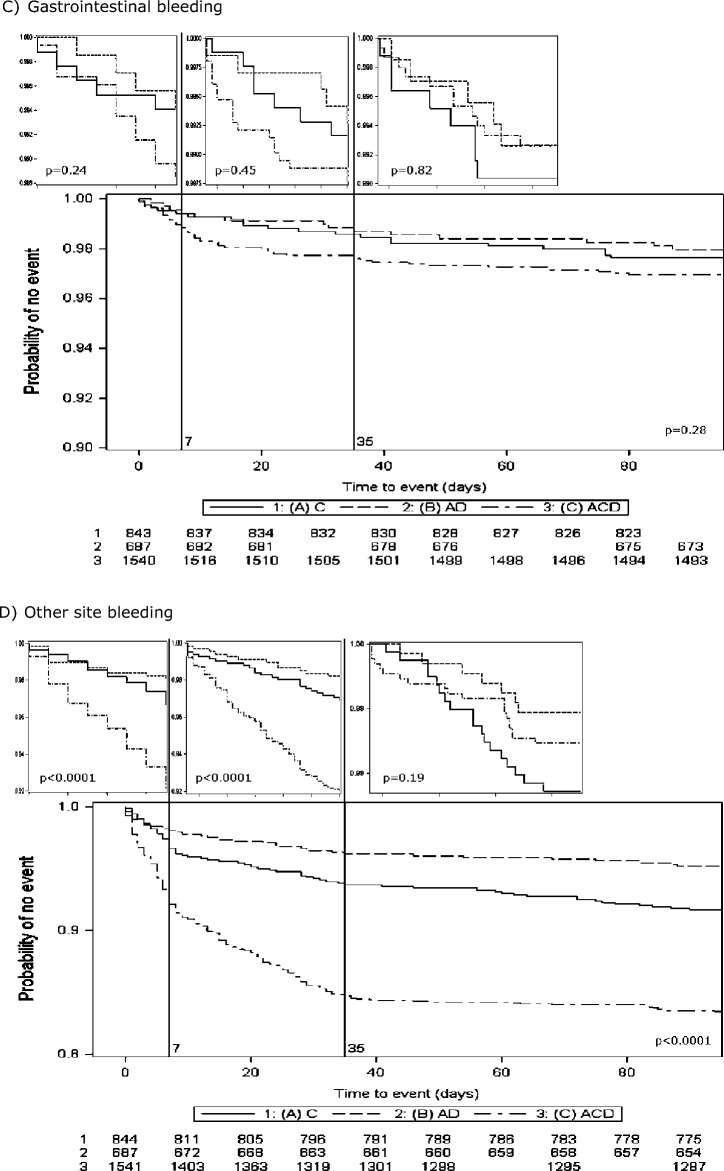
Time course of bleeding by antiplatelet regimen for epochs 0–7, 8–35 and 36–90 days. (**A**) Major bleeding; ACD: aspirin + clopidogrel + dipyridamole; AD: aspirin + dipyridamole; C: clopidogrel. (**B**) Intracranial bleeding; ACD: aspirin + clopidogrel + dipyridamole; AD: aspirin + dipyridamole; C: clopidogrel. (**C**) Gastrointestinal bleeding; ACD: aspirin + clopidogrel + dipyridamole; AD: aspirin + dipyridamole; C: clopidogrel. (**D**) Other site bleeding; ACD: aspirin + clopidogrel + di/958pyridamole; AD: aspirin + dipyridamole; C: clopidogrel.

There was a significant difference across the severity of bleeding events in regard to adherence to all of the first week of randomised treatment and for the S_2_TOP-BLEED score on treatment (Table 1). Across randomised treatment groups, bleeding events and their severity were significantly more common in those randomised to triple antiplatelet therapy compared to guideline therapy groups (Table 3, Fig. 1A). When the time course of bleeding was split into 0–7, 8–35 and 36–90 day epochs, the bleeding events diverged between the treatment groups in the 8–35 day period (*p* = 0.009) but not in the earlier or later periods. The time courses for intracranial and gastrointestinal bleeding did not differ by treatment group (Fig. 1B,C). In contrast, predominantly minor ‘other site’ bleeding events split early by treatment group and continued to diverge during the treatment period, then plateaued (Fig. 1).

Fatal or major bleeds were relatively infrequent occurring in 39 (2.5%) participants randomised to triple antiplatelet therapy, 12 (1.4%) randomised to clopidogrel, and 5 (0.7%) randomised to aspirin and dipyridamole. Major or fatal bleeding was increased with intensive vs. guideline antiplatelets: 39 vs. 17 participants, adjusted hazard ratio 2.21, 95% CI 1.24–3.93, *p* = 0.007. Performing subgroup analyses in this bleeding category was not feasible due to the small number of events. Increased numbers of intracranial and extracranial bleeds were seen in those randomised to triple antiplatelet therapy, but no significant associations were noted for composite outcomes ‘stroke or major haemorrhage’ or ‘major adverse cardiovascular event or major haemorrhage’ categories (Table 3).

### Ordinal bleeding and clinical outcomes

**Figure 2:**
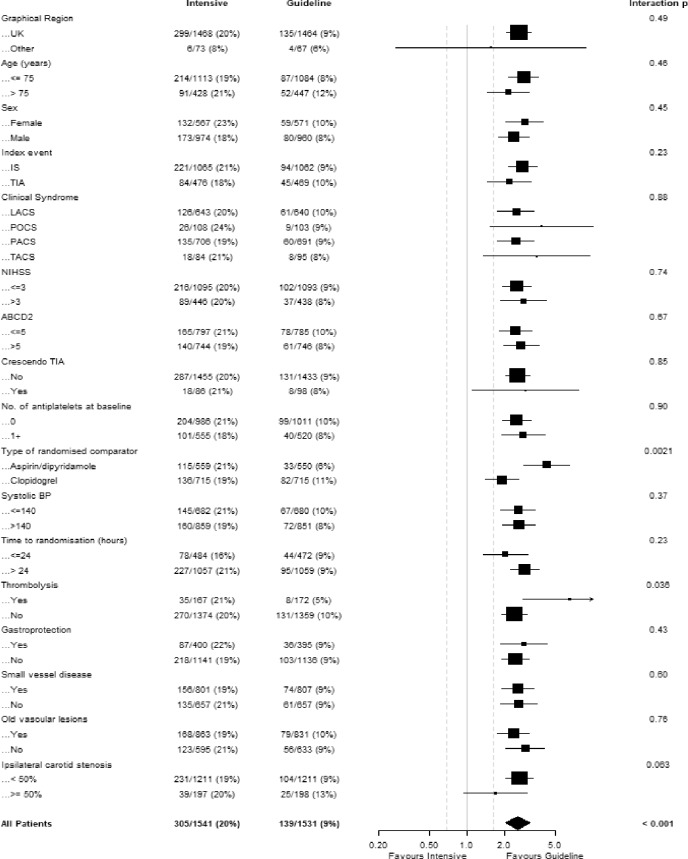
Forest plot of ordinal bleeding by randomised treatment (Intensive vs. guideline) ABCD2: age, blood pressure, clinical symptoms, diabetes, duration; BP: blood pressure; IS: ischaemic stroke; LACS: lacunar syndrome; NIHSS: National Institutes of Health Stroke Scale PACS: partial anterior circulation syndrome; POCS: posterior circulation syndrome; TACS: total anterior circulation syndrome; TIA: transient ischaemic attack.

More severe bleeding was associated with death or deterioration in hospital (Table 5). At day 90, more severe bleeding was associated with increased death and dependency (mRS), increased disability (Barthel Index), worse depression score (Zung) and worse quality of life scores (HSUV and EQ-VAS). When the treatment effect on ordinal bleeding was assessed in pre-specified subgroups, there was a statistically significant interaction with type of randomised guideline therapy; randomisation to aspirin + dipyridamole was associated with less and less severe bleeding than randomisation to clopidogrel (p for interaction = 0.0021). Similarly, those participants who underwent thrombolysis prior to enrolment randomised to triple antiplatelet therapy had more and more severe bleeding than those who did not receive thrombolysis (*p* for interaction = 0.036). Within all other pre-specified subgroups, there were no significant interactions with the treatment effect on ordinal bleeding (Fig. 2).

**Table 5:** Relationship between ordinal bleeding and outcomes in hospital and at 90 days.

	Bleeding^27^					
	Fatal	Major	Moderate	Minor	None	Trend *p*
Number of participants	11	45	38	350	2628	–
In hospital						
NIHSS, day 7 (/42) ‡	21.6 (21.3)	3.0 (5.2)	2.4 (3.2)	1.6 (2.4)	1.8 (3.8)	0.12
Death or deterioration	5 (45.5)	2 (4.4)	2 (5.3)	4 (1.1)	39 (1.5)	0.011
Length of stay in hospital [days]	1.0 [1.0, 6.0]	3.5 [0.0, 10.5]	2.0 [0.0, 12.0]	1.0 [0.0, 5.0]	1.0 [0.0, 5.0]	0.56
Died in hospital/discharged to institution (%)	5 (45.5)	4 (9.1)	4 (10.5)	19 (5.5)	169 (6.5)	0.42
Day 90						
mRS (/6) ‡	6.0 [6.0, 6.0]	2.0 [1.0, 3.0]	1.0 [1.0, 3.0]	1.0 [1.0, 2.0]	1.0 [0.0, 2.0]	< 0.001
BI (/100) ‡^29^	− 5.0 (0.0)	85.6 (28.3)	88.0 (24.1)	93.6 (17.3)	93.3 (18.2)	0.0023
ZDS (/102·5) ‡^34^	102.5 (0.0)	48.1 (18.0)	45.5 (15.1)	47.3 (16.4)	45.9 (17.0)	0.0027
t-MMSE (/23) ‡^39^	− 1.0 (0.0)	17.9 (5.6)	18.9 (1.8)	18.6 (3.7)	18.4 (4.2)	0.54
TICS-M (/37) ‡^31^	− 1.0 (0.0)	19.6 (7.4)	21.8 (3.7)	21.4 (5.7)	21.1 (6.2)	0.36
Verbal fluency ‡^32^	− 1.0 (0.0)	16.6 (8.4)	17.8 (7.1)	17.8 (7.3)	17.0 (7.5)	0.45
EQ-VAS (/100) ‡	− 1.0 (0.0)	64.9 (24.6)	66.8 (24.6)	73.6 (19.6)	72.7 (21.7)	0.031
EQ-5D3L-HSUV (/1) ‡^33^	0.0 (0.0)	0.6 (0.4)	0.7 (0.3)	0.7 (0.3)	0.8 (0.3)	0.0013
Death (%)	11 (100.0)	1 (2.2)	0 (0.0)	3 (0.9)	39 (1.5)	< 0.001

Data are number (percent), mean (standard deviation) and median [interquartile range].

Comparison by ANOVA, Kruskal–Wallis teat and Fisher’s exact test. BI: Barthel Index; EQ-5D3L-HSUV: EuroQol-5 dimensions 3 levels-health status utility values; EQ-VAS: EuroQol-Visual Analogue Scale; mRS: modified Rankin Scale; t-MMSE: modified telephone-Mini-Mental State Examination; NIHSS: National Institutes of Health Stroke Scale; TICS-M: Telephone Interview Cognition Scale-modified; ZDS: Zung Depression Scale; ‡: Death = NIHSS 43, mRS 6, BI -5, ZDS 102.5, mRS 6, t-MMSE -1, TICS-M -1, Verbal fluency -1, EQ-5D3L-HSUV 0, EQ-VAS -1.

### On-treatment bleeding scores

**Figure 3:**
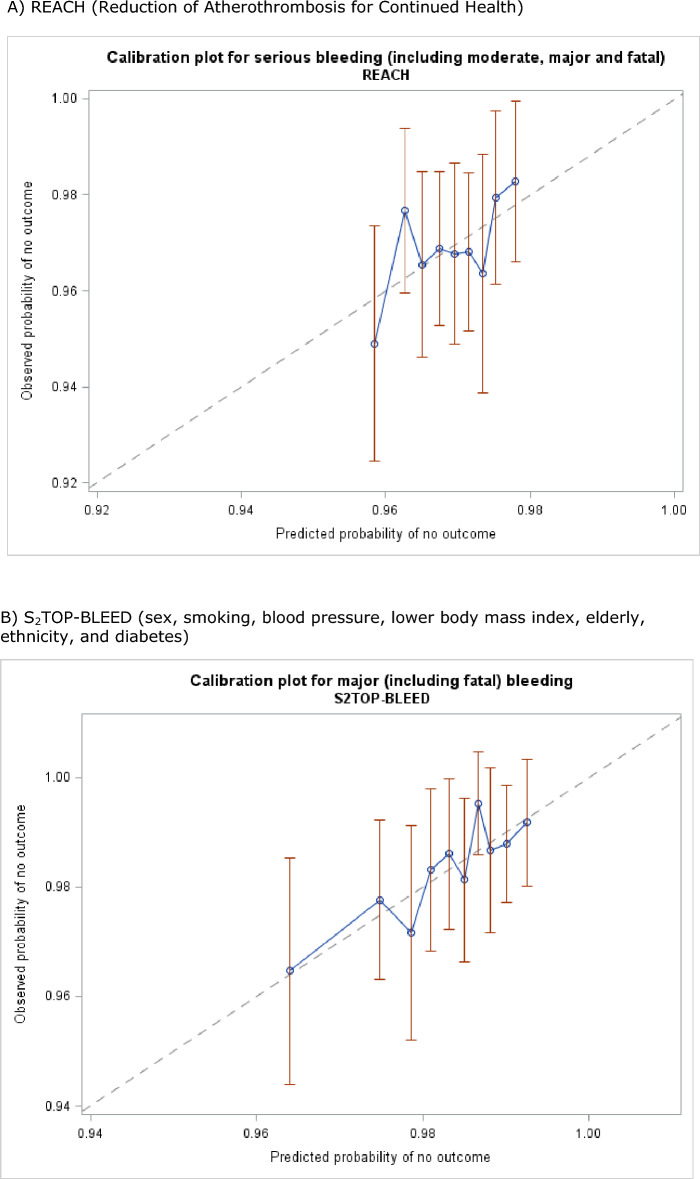

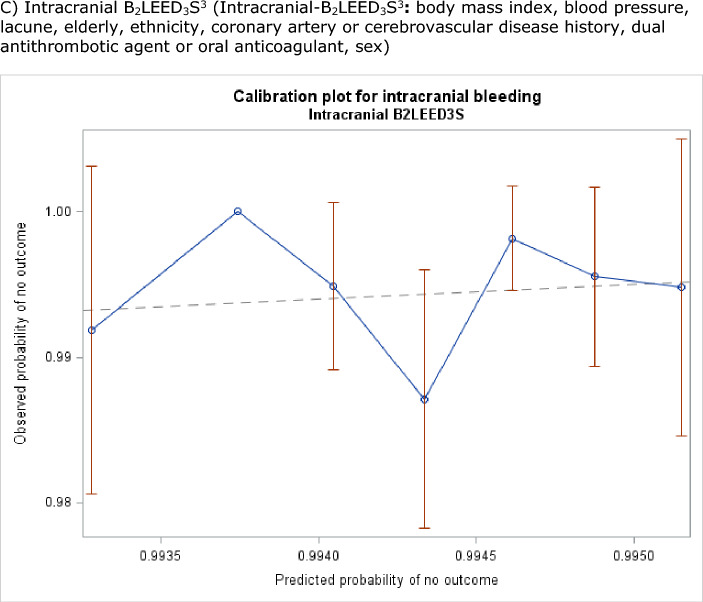
Calibration plots for bleeding-free survival for the three predictive scores on treatment. (**A**) REACH: Reduction of Atherothrombosis for Continued Health. (**B**) S_2_TOP-BLEED: sex, smoking, blood pressure, lower body mass index, elderly, ethnicity, and diabetes. (**C**) Intracranial B_2_LEED_3_S^3^: Intracranial-B_2_LEED_3_S^3^: body mass index, blood pressure, lacune, elderly, ethnicity, coronary artery or cerebrovascular disease history, dual antithrombotic agent or oral anticoagulant, sex.

The REACH score poorly predicted bleeding both by site and severity (Table 6, Fig. 3). The intracranial-B_2_LEED_3_S^3^ score poorly predicted intracranial bleeding in this population (C-statistic 0.51, 95% CI 0.39–0.64). In contrast, the S_2_TOP-BLEED score moderately predicted bleeding by severity and site. Specifically the S_2_TOP-BLEED score was designed to predict major, including fatal, bleeding and achieved this with a moderate C-statistic of 0.62 (95% CI 0.55–0.69) in those with fatal or major bleeding and 0.63 (95% CI 0.54–0.71) in those with a major bleed (Table 6, Fig. 3).

**Table 6:** C statistic (95% confidence intervals) for on-treatment REACH, S_2_TOP-BLEED and intracranial B_2_LEED_3_S^3^ bleeding scores^3–5^.

Bleeding	REACH	S_2_TOP-BLEED	Intracranial B_2_LEED_3_S^3^
Severity			
Fatal/Major	0.55 (0.47, 0.63)	0.62 (0.55, 0.69)	0.55 (0.48, 0.63)
Fatal	0.57 (0.38, 0.75)	0.62 (0.45, 0.79)	0.51 (0.36, 0.67)
Major	0.55 (0.47, 0.64)	0.63 (0.54, 0.71)	0.58 (0.49, 0.66)
Moderate	0.56 (0.48, 0.65)	0.63 (0.55, 0.71)	0.54 (0.46, 0.61)
Minor	0.51 (0.48, 0.54)	0.58 (0.55, 0.61)	0.50 (0.47, 0.54)
Any	0.52 (0.49, 0.55)	0.59 (0.56, 0.62)	0.50 (0.48, 0.53)
Site			
Intracranial	0.55 (0.42, 0.68)	0.65 (0.55, 0.76)	0.51 (0.39, 0.64)
Gastrointestinal	0.51 (0.45, 0.57)	0.57 (0.50, 0.63)	0.52 (0.45, 0.58)
Epistaxis	0.51 (0.44, 0.57)	0.55 (0.48, 0.61)	0.51 (0.44, 0.58)
Genitourinary	0.58 (0.50, 0.65)	0.64 (0.54, 0.73)	0.56 (0.47, 0.64)
Cutaneous	0.50 (0.46, 0.54)	0.58 (0.54, 0.62)	0.52 (0.47, 0.56)
Other	0.58 (0.50, 0.65)	0.59 (0.52, 0.66)	0.51 (0.43, 0.59)

Intracranial-B_2_LEED_3_S^3^: body mass index, blood pressure, lacune, elderly, ethnicity, coronary artery or cerebrovascular disease history, dual antithrombotic agent or oral anticoagulant, sex; REACH: Reduction of Atherothrombosis for Continued Health; S_2_TOP-BLEED: sex, smoking, blood pressure, lower body mass index, elderly, ethnicity, and diabetes.

## Discussion

In this large trial assessing triple versus guideline antiplatelet therapy in acute non-cardioembolic ischaemic stroke or TIA, fatal or major bleeding occurred more frequently in those randomised to triple vs guideline antiplatelet therapy. The divergence in major bleeding between the treatment groups occurred in the 8–35 day period but not in the earlier 0–7 and later 36–90 day periods. In multivariate analysis more, and more severe, bleeding events were seen with increasing age, female sex, increasing pre-morbid dependency, longer time from onset to randomisation, prior major bleed, prior antiplatelet therapy and in those randomised to triple vs guideline antiplatelet therapy. More severe bleeding was associated with worse clinical outcomes across multiple physical, emotional and quality of life domains. Those patients who underwent thrombolysis prior to enrolment had more, and more severe, bleeding in the presence of triple than guideline antiplatelet therapy. Of the bleeding scores assessed, the S_2_TOP-BLEED score moderately predicted major or fatal bleeding, whilst REACH and intracranial-B_2_LEED_3_S^3^ scores performed poorly.

The Platelet-Oriented Inhibition in New TIA and minor ischemic stroke (POINT)^6^ and the Clopidogrel in High-risk patients with Acute Nondisabling Cerebrovascular Events (CHANCE)^7^ trials both assessed aspirin plus clopidogrel vs aspirin alone in mild ischaemic stroke and TIA within 12 and 24 h of symptom onset, respectively. There were fewer bleeding events in both these trials compared with TARDIS. Major bleeding in POINT was seen in 0.9% of the intensive group (aspirin and clopidogrel) and 0.6% in the placebo (aspirin) group^6^, whilst CHANCE reported moderate or severe bleeding in 0.3% of patients in both treatment arms of the trial^7^. A recent systematic review and meta-analysis including four trials of dual antiplatelet therapy vs aspirin (CHANCE, POINT, THALES (The acute stroke or transient ischaemic attack treated with Ticagrelor and aspirin for prevention of stroke and death) and FASTER (Fast assessment of stroke and transient ischaemic attack to prevent early recurrence) found that dual antiplatelet therapy was associated with an increased risk of moderate or severe bleeding: relative risk 2.17, 95% CI 1.16 to 4.08)^8^. The authors stratified bleeding risk by treatment regimen or duration and noted the association was mainly seen with ticagrelor and treatment over 21 days.^8^ In contrast to TARDIS, dual antiplatelet therapy was not associated with an increased risk of minor bleeding events^8^. In addition to the number of antiplatelet agents being assessed, TARDIS differed from these studies by recruiting patients within 48 h of onset with no upper stroke severity limit and included 341 (11%) patients who had undergone thrombolysis. These differences, and the intensity of antiplatelet therapy, likely explain the higher bleeding rates seen in TARDIS, although it is interesting to note that stroke severity (NIHSS) was not associated with bleeding. The median [interquartile range] baseline NIHSS was 2.0 [1.0, 4.0] in TARDIS, compared with 2.0 [1.0, 3.0] in CHANCE^7^, 2.0 [1.0, 2.0] in POINT^6^, and 1.0 in FASTER^9^. THALES included patients with NIHSS ≤ 5: 60% NIHSS 0–3, 30% NIHSS 4–5^10^. In a secondary analysis of THALES comparing those with NIHSS 0–3 to NIHSS 4–5, there was no difference in the treatment effect with Ticagrelor-Aspirin vs Aspirin alone, and no difference in severe bleeding^11^. Future research may wish to consider whether dual antiplatelet therapy in more severe stroke populations reduces recurrent ischaemic events without causing excessive bleeding.

Multivariate associations between baseline variables and ordinal bleeding revealed that increasing age, female sex, increasing pre-morbid dependency, a previous major bleed, previous antiplatelet therapy, and a longer time from onset to randomisation were associated with more severe bleeding events. In contrast, a secondary analysis of POINT reported that the only baseline variable significantly associated with the risk of major haemorrhage was increasing age^12^. In TARDIS, 70% of participants were recruited 24-48 h after onset, which is a high risk period for blood–brain barrier breakdown due to ischaemia, and coupled with triple antiplatelet therapy likely increased the risk of bleeding. Despite having pre-morbid mRS > 2 as an exclusion criteria, increasing pre-morbid mRS was associated with more severe bleeding events. Imaging markers of ‘brain frailty’, both individually and when amalgamated as a score, were not associated with increased bleeding although cerebral atrophy narrowly missed statistical significance. In a secondary analysis of the third international stroke trial (IST-3), cerebral atrophy (but not other background changes) was associated with an increased risk of symptomatic intracranial haemorrhage (sICH)^13^, whilst a secondary analysis of the enhanced control of hypertension and thrombolysis stroke study (ENCHANTED) found no association between atrophy and sICH^14^. In TARDIS, background imaging markers were surprisingly prevalent in a population who were independent according to the mRS at baseline. Clinical frailty was not measured in TARDIS but the burden of ‘brain frailty’ may suggest that this cohort was frailer than perhaps the mRS is able to delineate, which may have influenced the association with bleeding events.

In the present study, the only bleeding score to moderately predict bleeding for which it was designed was the S_2_TOP-BLEED score for major, including fatal, bleeding. This finding is similar to an external validation study from the Oxford Vascular Study group where the S_2_TOP-BLEED score was better at predicting bleeding for which it was designed, than the REACH and intracranial-B_2_LEED_3_S^3^ scores in 2072 patients on an antiplatelet agent following a TIA or ischaemic stroke^15^. Future trials may wish to stratify patients by bleeding risk in order to reduce the potential risk of bleeding in this patient group.

TARDIS used an ordinal bleeding scale^16^ incorporating minor bleeding events, which were more common in the intensive than guideline group, occurring from treatment onset throughout the treatment period. In contrast, major bleeding events occurred later in the treatment period. Meta-analyses of dual antiplatelet therapy trials have demonstrated that a short period (21 days) of intensive antiplatelet therapy seems to be optimum; reducing the risk of major bleeding events, whilst maximising any treatment benefit^8,17^. Whether an even shorter period of intensive triple antiplatelet therapy for 7 days is safe and effective is unclear.

This secondary analysis of TARDIS has several strengths including wide inclusion criteria and therefore generalisability, use of locally-sourced antiplatelet agents from a variety of manufacturers increasing validity, and an ordinal bleeding safety outcome incorporating minor bleeding. However, there are several limitations. First, the wide inclusion criteria included no upper stroke severity limit and allowed inclusion of patients up to 48 h after symptom onset. Although this allowed generalisability, increasing time to randomisation was associated with increased bleeding. Second, patients could be recruited 24–48 h after thrombolysis; hence, TARDIS shows, for the first time in a large trial, that acute intensive antiplatelet therapy should not be administered to patients 24–48 h after thrombolysis. Third, trial treatments were open label and therefore participants knew what they were taking and may have been more likely to report bleeding events, especially minor bleeds. Last, 30 days of triple treatment may have been too long given the associated bleeding risk and that bleeding events diverged between treatment groups in the 8–35 day period, but not before or after. Hence, the effect on bleeding occurred whilst on treatment and not after. The lack of divergence in the 0–7 day period implies that the duration of intensive antiplatelet therapy likely contributes to the risk of bleeding as demonstrated in the aforementioned meta-analysis^8^.

Further research, including meta-analysis of trials, should seek to establish the optimum timing of initiation, duration and types of antiplatelet therapy in acute minor ischaemic stroke and TIA. Triple antiplatelet therapy should be avoided in acute ischaemic stroke and TIA and particularly 24–48 h following thrombolysis pending further data. Stratifying patients using bleeding scores may help to minimise bleeding risk and rationalise antiplatelet treatment in future studies.

In summary, triple antiplatelet therapy in acute non-cardioembolic ischaemic stroke and TIA patients was associated with more fatal or major bleeds and more, and more severe, bleeding. For major bleeding events, the treatment groups separated after one week supporting the rationale for a short period of intensive treatment in dual antiplatelet trials of minor ischaemic stroke and TIA patients to maximise benefit whilst minimising bleeding risk. Increasing age, female sex, increasing pre-morbid dependency, longer time from onset to randomisation, previous major bleed and prior antiplatelet therapy were all associated with more, and more severe, bleeding. Clinicians should take these factors into consideration when counselling patients with acute cerebral ischaemia on the bleeding risk with intensive antiplatelet therapy.

## Methods

### Study design

TARDIS was an international multicentre prospective randomised open-label blinded-endpoint (PROBE) trial conducted in four countries (Denmark, Georgia, New Zealand, UK) at 106 sites. Information on the design, statistical analysis plan and baseline characteristics of the participants have already been published^18–20^.

Adult patients aged ≥ 50 years were suitable for inclusion if they were at risk of a recurrent ischaemic stroke and had either a non-cardioembolic ischaemic stroke with limb weakness, isolated dysphasia or isolated neuroimaging-positive hemianopia, or a non-cardioembolic TIA with at least 10 min of limb weakness or isolated dysphasia. Participants were randomised within 48 h of the onset of symptoms; if they had received intravenous thrombolysis they could be randomised after 24 h following receiving this treatment and brain imaging had excluded any cerebral bleeding. There was no stroke severity cut-off for inclusion into the trial. Exclusion criteria included: isolated sensory symptoms, facial weakness, vertigo or dizziness; intracranial haemorrhage; non-ischaemic cause for symptoms; definite need for, or contraindication to, aspirin, clopidogrel or dipyridamole; need for anticoagulation; and pre-randomisation dependency. Patients provided written informed consent, or if the patient lacked capacity written informed consent was obtained from a relative, carer or friend. A full listing of the study inclusion and exclusion criteria is available^1^.

### Interventions

Randomisation was provided by entering data online into a secure web-based database system. Treatment assignment was stratified by country and index event (stroke, TIA), with minimisation on key prognostic baseline factors^1^ Participants randomised to the intervention group received aspirin (300 mg loading followed by 50-150 mg daily, typically 75 mg), clopidogrel (300 mg loading followed by 75 mg daily) and dipyridamole (200 mg twice daily modified release). Those randomised to guideline antiplatelet therapy received either clopidogrel alone or combined aspirin and dipyridamole, using the same doses above^1^ Randomised treatment was given for 30 days after which participants were treated according to local guidelines.

### Assessments

Age, sex, ethnicity and prior antiplatelet therapy (none, aspirin, clopidogrel, dipyridamole, other) were recorded at baseline. The severity of index stroke was assessed with the National Institutes of Health Stroke Scale (NIHSS, scores range from 0 to 42 with higher scores indicating a more severe neurological deficit^21^) and risk of recurrence after index TIA assessed using the ABCD2 scale (scores range from 0 to 7 with higher scores indicating a higher risk of recurrence^22^). Clinical syndrome was assessed using the Oxfordshire Community Stroke Project classification^23^.

Participants were seen at days 7 (on treatment) and 35 (end of treatment) to determine whether any outcome or bleeding events had taken place and to assess treatment compliance. Final follow-up was performed centrally by blinded assessors at 90 days by telephone from the coordinating centre in each country. If the participant could not be contacted, a paper version of the form was sent by post. Data pertaining to recurrent cerebrovascular events were captured at days 7 and 35 (investigator-reported), in any serious adverse events, at day 90 telephone follow-up and in a general practitioner questionnaire after day 90. Expert adjudicators blinded to treatment assignment validated and categorised the primary outcome, haemorrhage and investigator-reported serious adverse events. Any participants who violated the protocol were still followed up in full and included in intention-to-treat analyses.

### Neuroimaging

Brain imaging was essential for all ischaemic stroke patients prior to enrolment in TARDIS in order to exclude haemorrhage or an alternative imaging diagnosis. Imaging was not essential for patients with TIA. Computerised topography (CT) or magnetic resonance imaging (MRI) scans performed according to local practice and any additional clinical scans were collected and adjudicated centrally by a trained panel of expert neuroradiologists blinded to symptoms and randomised treatment, using approaches developed from the third international stroke trial (IST-3) and the efficacy of nitric oxide in stroke (ENOS) trial^13,24^.

Acute stroke lesions were graded by location, size, severity, swelling and mass effect. The Alberta Stroke Program Early CT Score (ASPECTS) was used to grade the degree of ischaemia affecting the middle cerebral artery territory.

Baseline pre-event imaging markers including cerebral atrophy, periventricular white matter lucencies and old vascular lesion(s) were assessed individually, and amalgamated as a ‘brain frailty’ score^25^.

- Cerebral atrophy: assessed in cortical and central regions as 0 = absent, 1 = moderate, 2 = severe.
- Periventricular white matter lucencies: assessed in anterior and posterior regions as 0 = absent, 1 = lucency restricted to region adjoining ventricles, 2 = lucency covering lateral ventricle to cortex.
- Old vascular lesions: assessed by location.
- ‘Brain frailty’ score: 1 point for periventricular white matter lucencies (score of 1 or 2 anterior and/or posterior); 1 point for cerebral atrophy (scores of 1 or 2 cortical and/or central); and 1 point for old vascular lesion(s) (maximum 3 of 3).

### Bleeding and other outcome measures

The severity of bleeding was assessed on a five level ordered categorical scale^16,26^: fatal, major, moderate, minor, none. The definitions of fatal, major and moderate haemorrhage were according to the International Society on Thrombosis and Haemostasis and are based on severity, bleeding site, haemoglobin drop and transfusion requirement^27^.

Further safety outcomes included all-cause and cause-specific case fatality, early neurological deterioration (defined as an increase from baseline to day 7 of at least 4 points on the NIHSS and/or a reduction in consciousness in the NIHSS consciousness domain), and serious adverse events.

The primary efficacy outcome was measured at 90 days and comprised stroke/TIA recurrence and its severity; severity was assessed using a six-level ordinal scale^19,26^: fatal stroke, non-fatal severe stroke (modified Rankin Scale, mRS 4, 5), moderate stroke (mRS 2, 3), mild stroke (mRS 0, 1), TIA, and neither stroke nor TIA^18,19^. Day 90 secondary outcomes^28^ included activities of daily living (Barthel Index^29^); cognition (telephone Mini-Mental State Examination, t-MMSE^30^; Telephone Interview for Cognition Scale-modified, TICS-M^31^; verbal fluency^32^); health-related quality of life (European Quality of Life-5 dimensions-3 level, EQ-5D-3L^33^, from which health status utility values, HSUV, were calculated; and EQ-visual analogue scale, EQ-VAS); and mood (short Zung Depression Score ^34^). At the time of hospital discharge, the length of hospital stay and discharge destination were recorded.

### Bleeding risk prediction scores

Bleeding risk prediction scores were calculated from information provided at baseline as outlined in Supplementary Table 1. The REACH (Reduction of Atherothrombosis for Continued Health) score was designed to predict serious bleeding (including moderate, major and fatal)^3^. The S_2_TOP-BLEED score predicts major (including fatal) bleeding and incorporates sex, smoking, blood pressure, lower body mass index, elderly, ethnicity and diabetes in the algorithm^4^. The B_2_LEED_3_S^3^ score predicts intracranial bleeding and incorporates sex, body mass index, blood pressure, lacune, elderly, ethnicity, coronary artery or cerebrovascular disease history and dual antithrombotic agent or oral anticoagulant in the algorithm^5^.

### Study oversight

All methods were carried out in accordance with relevant guidelines and regulations. All experimental protocols were approved by the East Midlands UK research ethics committee (reference: 08/H1102/112). The study was registered (ISRCTN47823388, EudraCT: 2007-006749-42) and adopted in the UK by the National Institute of Health Research (NIHR) Stroke Research Network. National competent authority (or equivalent) approvals were also obtained for this study. The study was sponsored by the University of Nottingham, UK.

### Statistical analyses

Data are number (%), median [interquartile range] or mean (standard deviation).The effect of treatment on the primary safety outcome was analysed as a shift in bleeding and its severity (fatal, major, moderate, minor and none) using ordinal logistic regression. Additional sensitivity analyses included assessing bleeding individually by level of severity and as all bleeding. Comparisons between bleeding severity levels and treatments were assessed using ANOVA, the Chi-Square/Fisher's Exact test, the Kruskal-Wallis test, Kendall's rank correlation and Jonckheere-Terpstra test. Further analyses were performed using binary logistic regression (reported as adjusted odds ratio, aOR), ordinal logistic regression (adjusted common odds ratio, aOR) or multiple linear regression (adjusted mean difference, aMD); 95% confidence intervals are given with each, and covariates comprised: index event (TIA vs stroke), country, guideline randomisation choice, age, sex, pre-morbid function, systolic blood pressure, stroke syndrome (cortical vs. lacunar^23^), number of antiplatelets before index event, use of gastroprotection, use of low dose heparin, time to randomisation, NIHSS, ABCD2 score, number of TIAs in the last week and treatment with alteplase. A check that the assumption of common proportional odds was not violated was performed before ordinal logistic regression using the likelihood ratio test.

To assess the treatment effect on ordinal bleeding in pre-specified subgroups, an interaction term was added to an adjusted ordinal logistic regression model. Clinical outcomes included a value for death, as is standard for the mRS, Barthel Index and EQ-5D; values comprised: mRS 6, Barthel Index -5, t-MMSE -1, TICS-M -1, Verbal fluency -1, EQ-5D3L-HSUV 0, EQ-VAS -1, Zung 102.5. Age was analysed using a pre-specified cut-point (≤ 75 vs > 75 years) as well as a continuous variable using multiple linear regression.

We compared performance of the REACH, S_2_TOP-BLEED and intracranial B_2_LEED_3_S^3^ bleeding risk scores^3–5^ by means of the C statistic for integrated discrimination improvement, and calibration plot analysis^35–37^. The nominal level of significance for all analyses was *p* < 0.05. No adjustment was made for multiplicity of testing for secondary analyses. All analyses were by the intention to treat principle for all comparisons. Statistical analyses were performed by LJW using SAS software (version 9.4).

## Supplementary Information

Supplementary Information.

## Data Availability

The datasets used during the current study are available from the corresponding author on reasonable request. Data have been shared with the virtual international stroke trials archive (VISTA): https://www.virtualtrialsarchives.org/vista/.
