# Supplementary material for: Bleeding with intensive versus guideline antiplatelet therapy in acute cerebral ischaemia
Source: Sci Rep. 2023 Jul 20;13:11717. doi: 10.1038/s41598-023-38474-2 (PMC10359249; doi:10.1038/s41598-023-38474-2)
Supplement: Supplementary file 1 — Supplementary Information. [file 41598_2023_38474_MOESM1_ESM.pdf]

Bleeding with intensive versus guideline antiplatelet therapy in acute cerebral ischaemia

SUPPLMENTARY INFORMATION

Supplementary Table 1: Bleeding score components

REACH	S_2TOP-BLEED	Intracranial $B_2LEED_3S^3$
Age	Sex	Body mass index
Peripheral arterial disease	Smoking status	Hypertension
Congestive heart failure	Antiplatelet use	TOAST criteria (small vessel disease)
Diabetes	Pre-morbid mRS	Age
Hypercholesterolaemia	Prior stroke	Ethnicity
Hypertension	Hypertension	Cardiovascular disease
Smoking status	Body mass index	Cerebrovascular disease
Antiplatelet / anticoagulant use	Age	Antithrombotic / anticoagulant use
	Ethnicity	Sex
	Diabetes	

The TARDIS Investigators

A complete list of Investigators is provided in the Supplementary Appendix of the main publication (reference 1 in accompanying manuscript).

Writing Committee

Lisa J Woodhouse, Jason P Appleton, Hanne Christensen, Rob A Dineen, Timothy J England, Marilyn James, Kailash Krishnan, Alan A Montgomery, Anna Ranta, Thompson Robinson, Nikola Sprigg; Philip M Bath, for the TARDIS Investigators

Trial Steering Committee
Independent members: Helen Rodgers (Newcastle, TSC Chair), Ahamad Hassan (Leeds), Christine Roffe (Stoke-on-Trent), Craig Smith (Salford), William D Toff (Leicester)
Grant holders: Philip Bath (Nottingham, Chief Investigator), Rob Dineen (Nottingham, Neuroradiology Lead), Lelia Duley (Nottingham), Stan Heptinstall (Nottingham, Platelet Expert), Marilyn James (Nottingham, Health Economic Lead), Hugh Markus (Cambridge), Stuart Pocock (London, Statistical Lead), Thompson Robinson (Leicester), Nikola Sprigg (Nottingham, Deputy Chief Investigator), Graham Venables (Sheffield)
Patient-public representative (Nottingham): Oswald Newell (2008-14), Chibeka Kasonde (2014-16)
Sponsor's representative: Angela Shone (University of Nottingham)

International Advisory Committee
Denmark - Hanne Christensen (Copenhagen), Georgia - Maia Beridze (Tblisi), New Zealand - Anna Ranta (Wellington), UK – Philip Bath (Chair, Nottingham)

Independent Data Monitoring Committee
Ian Ford (Glasgow, UK; Chair), Didier Leys (Lille, France), Cathie Sudlow (Edinburgh, UK), Matthew Walters (Glasgow, UK)

Events (outcome, SAE) Adjudicators
Nikola Sprigg (Stroke Physician, Nottingham, UK), Marc Randall (Neurologist, Leeds, UK), Wayne Sunman (Stroke Physician, Nottingham, UK), Kailash Krishnan (Stroke Physician, Nottingham, UK)

Neuroimaging Adjudicators
Rob Dineen (Nottingham, UK), Alessandro Adami (Verona, Italy), Lesley Cala (Perth, Australia), Ana Casado (Edinburgh, UK), Rebecca Gallagher (Derby, UK), David Swienton (Leicester, UK), Satheesh Ramalingam (Birmingham, UK)

Platelet substudy
Stan Heptinstall, Sue Fox, Jane May (Nottingham, UK)
